# Bioinspired Twisted Artificial Muscles with Enhanced Performance for Underwater Applications

**DOI:** 10.1002/advs.202507572

**Published:** 2025-07-11

**Authors:** Jin Sun, Yuan Fu, Shijng Zhang, Jing Li, Dehong Wang, Junkao Liu, Yingxiang Liu

**Affiliations:** ^1^ State Key Laboratory of Robotics and Systems Harbin Institute of Technology Harbin Heilongjiang Province 150001 China

**Keywords:** braided‐twisted and plant‐coiled artificial muscles, rapid actuation unit, soft actuator, thermal insulation strategy, underwater application

## Abstract

Twisted artificial muscles (TAMs) demonstrate great promise in robotic applications involving locomotion and manipulation. However, their functionality in underwater environments remains challenging due to limitations in deformation, output force, and heat dissipation especially for thermally driven TAMs. To address these challenges, a novel TAM configuration inspired by the twining structures of climbing plants that consists of braided and pre‐twisted fiber bundles is proposed. This configuration achieves large deformation and high output force, reaching a contraction ratio of 40.0% under a load of 300 g. Meanwhile, a soft insulation layer inspired by the blubber layer of seals is applied to reduce heat dissipation in underwater environments, resulting in a 30.5 °C temperature difference. In addition, a rapid actuation unit is developed, which utilizes elastic energy storage and release to achieve an angular velocity of 180° s^−1^ in water. Finally, a bionic ray driven by the proposed TAMs is developed as a demonstrator, achieving a displacement of 105 mm for straight motion and a turning angle of 30° within a single actuation cycle. These results highlight the strong potential of the proposed TAMs for underwater application.

## Graphical abstract

A climbing plant‐inspired twisted artificial muscle (TAM) with a braided configuration and seal‐inspired thermal insulation enables high‐performance underwater actuation. Enhanced deformation, reduced heat dissipation, and rapid motion are validated through a bionic ray that exhibits straight and turning motions. This work highlights the potential of TAMs for underwater applications.

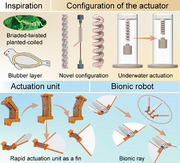

## 1 Introduction

Twisted artificial muscles (TAMs) represent a class of soft actuators that exhibit substantial tensile and contractile deformation. Compared to other soft actuators such as dielectric elastomer actuators (DEAs),^[^ ^1^, ^2^, ^3^, ^4^, ^5^ ^]^ shape memory alloys (SMAs),^[^ ^6^, ^7^, ^8^ ^]^ electro fluidic actuators (EFAs),^[^ ^9^, ^10^, ^11^ ^]^ triboelectric nanogenerators (TENGs),^[^ ^12^, ^13^, ^14^ ^]^ and pneumatic actuators (PAs),^[^ ^15^, ^16^, ^17^, ^18^, ^19^ ^]^ TAMs are distinguished by their low cost and ease of fabrication.^[^ ^20^, ^21^, ^22^, ^23^, ^24^, ^25^, ^26^, ^27^ ^]^ Additionally, they support diverse actuation methods, including electrochemical, thermal, and solvent‐driven approaches. These advantages make TAMs highly adaptable to various robotic applications.

In recent years, TAMs have been employed to drive soft robots with multifunctional capabilities, enabling locomotion^[^ ^28^, ^29^, ^30^, ^31^, ^32^ ^]^ and manipulation.^[^ ^33^, ^34^, ^35^, ^36^, ^37^, ^38^, ^39^, ^40^ ^]^ For example, for locomotion tasks, Zhou et al.^[^ ^29^ ^]^ proposed a tensegrity robot with a load capacity of 1.5 N and a speed of 0.17 mm s⁻¹. Tang et al.^[^ ^41^ ^]^ developed a soft crawling robot driven by a single TAM, with a straight motion speed of 1.2 mm s⁻¹. Similarly, Hu et al.^[^ ^42^ ^]^ presented a modular robot that could crawl in a pipe with a speed of 0.13 mm s⁻¹. The low speed of these robots mainly results from their limited deformation and low actuation frequency. For manipulation tasks, Sun et al.^[^ ^39^ ^]^ proposed a miniature surgical robot capable of achieving precise motion with a maximum bending angle of 90°. Although the performance of TAM‐driven robots still requires improvement, their fundamental capabilities have been demonstrated. However, the underwater applications of TAMs have received little attention, especially considering that 71% of our planet's surface is covered by water.^[^ ^43^, ^44^, ^45^ ^]^

The applicability of TAMs in underwater environments primarily depends on their actuation methods. For instance, electrochemical‐driven TAMs require an electrolyte system, whose additional volume and weight make them unsuitable for underwater environments. Similarly, solvent‐driven TAMs^[^ ^21^, ^46^, ^47^ ^]^ contract by absorbing water vapor, but the inability to dry in underwater environments prevents continuous actuation. In contrast, thermal‐driven TAMs^[^ ^48^, ^49^ ^]^ can achieve a compact structure by simultaneously twisting fibers and electric heating wires, making underwater actuation possible. However, it is crucial to maintain a sufficient temperature and ensure that satisfactory deformation and output force can be achieved at relatively low temperatures to realize the successful underwater operation of thermal‐driven TAMs. Specifically, Xiang et al.^[^ ^50^ ^]^ proposed a method to enhance output force by co‐winding SMA with nylon fibers, but it achieved only 8% contraction under a load of 300 g. Noh et al.^[^ ^33^ ^]^ introduced an Ag nanoparticles‐based twisted and coiled actuator with a contractile actuation of 36%, while the load force is only 0.7 N.

**Figure 1:**
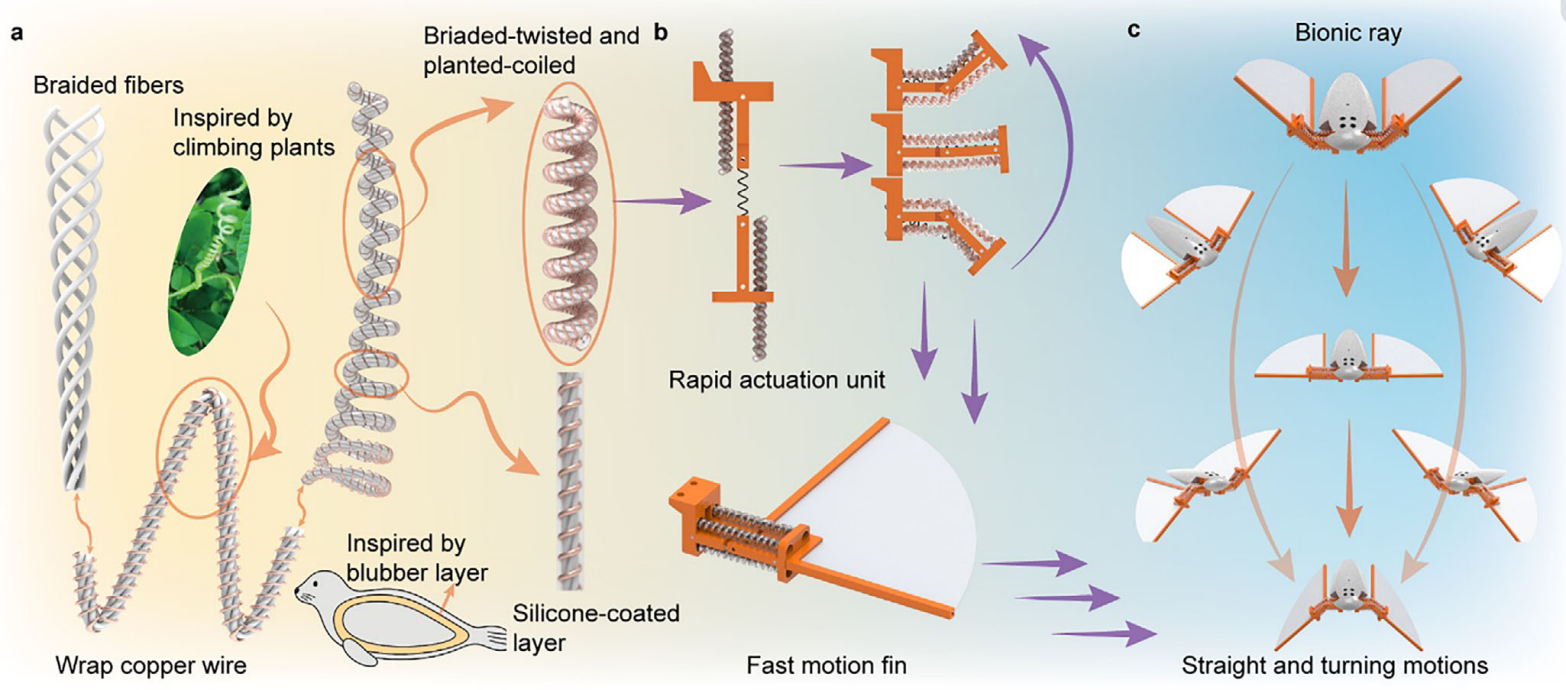
Structure and underwater applications of SBPAM. a) The novel configuration of BPAM, inspired by the climbing morphology of plants, exhibits both large deformation and high output force; the soft thermal insulation layer, inspired by the blubber layer of seals, is applied to reduce heat dissipation in water. b) Schematic of the RAU, which consists of four SBPAMs, two pedestals, an energy storage spring, and fixation rods. The soft fins are securely integrated with the RAUs, enabling passive, wave‐like deformation. c) A bionic ray robot driven by SBPAMs is developed, capable of performing both straight and turning motions in underwater environments.

To address these limitations and evaluate the performance of thermal‐driven TAMs for underwater applications, the main contributions of this work are as follows: 1) A novel TAM configuration inspired by climbing plants is proposed, named as braided‐twisted and plant‐coiled artificial muscle (BPAM), achieving both large deformation and high output force (40.0% contraction under a load of 300 g), as shown in **Figure** **1**a. 2) A thermal insulation strategy inspired by the blubber layer of seals is applied to construct silicone‐coated braided‐twisted and plant‐coiled artificial muscle (SBPAM), which effectively reduces underwater heat dissipation, resulting in a 30.5 °C temperature difference between the center of TAM with and without blubber layer. 3) A rapid actuation unit (RAU) is developed, which utilizes the storage and release of elastic energy to achieve an angular velocity of 180° s⁻¹, substantially improving the actuation speed to overcome the water resistance, as shown in Figure 1b. 4) A bionic ray is presented as a demonstrator, achieving straight and turning motions, as shown in Figure 1c. The experimental results confirm that the proposed configuration of TAMs exhibits significant potential for underwater applications.

## 2 Results

### 2.1 Configuration Design of the BPAM with Large Deformation and High Output Force

Underwater applications of TAMs require both large deformation and high output force, yet achieving this balance remains challenging. To explore this issue, we develop a theoretical model that elucidates the relationship between structural parameters and actuation performance (see Figure S1 and Note S1, Supporting Information). The analysis reveals that the contraction ratio is primarily proportional to the untwisting coefficient *c* which describes the angular untwisting per unit fiber length induced by thermal activation, as well as the mean diameter *D* and the temperature rise *ΔT*, while the output force scales with the diameter of the fiber. Drawing inspiration from the helical climbing patterns of vine plants, the fibers are individually pre‐twisted and then twisted together to achieve a higher untwisting coefficient, and we enhance the spring index of the helical structure to increase the mean diameter of BPAM. Together, these modifications result in a marked improvement in contraction performance. In addition, a braided configuration is employed to increase the equivalent diameter and stiffness, thereby enhancing the output force. These combined strategies collectively enable a balanced improvement in both deformation and output force, thereby fulfilling the comprehensive performance requirements for underwater actuation.

**Figure 2:**
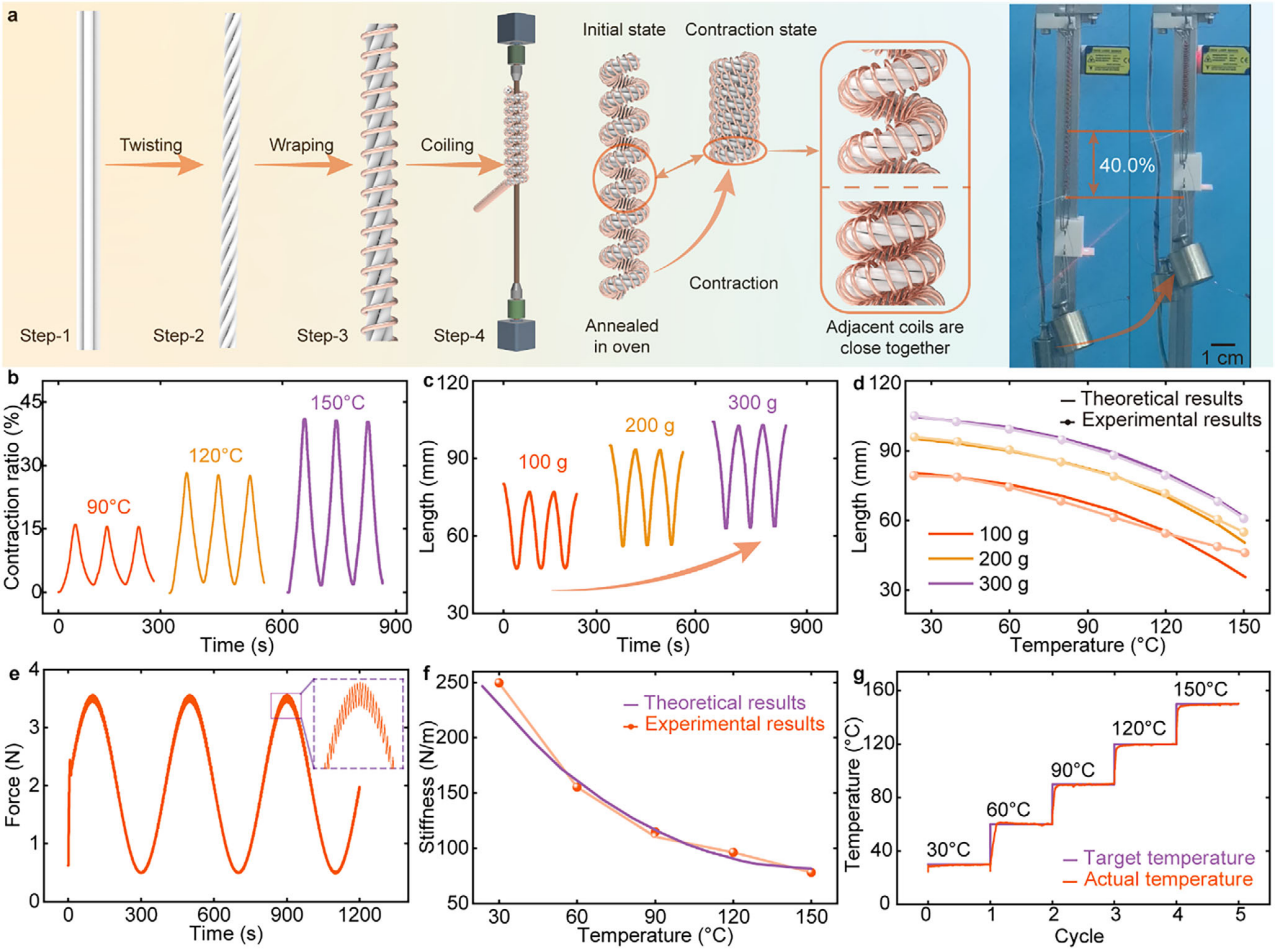
Configuration, characterization, and experimental results of BPAM. a) Fabrication process and prototype of BPAM. b) Contraction ratio of BPAM under set temperatures of 90 °C, 120 °C, and 150 °C. c) Length variation of BPAM under loads of 100, 200, and 300 g; a contraction ratio of 40.0% is achieved under a 300 g load. d) Comparison between theoretical predictions and experimental results for length change with increasing temperature. e) Output force control performance of BPAM over a range from 0.5 N to 3.5 N. f) Comparison of theoretical and experimental stiffness values of BPAM. g) Target and actual temperatures during stiffness measurement tests.

The fabrication process of the BPAM is illustrated in **Figure** **2**a and consists of five steps. Initially, four single fibers are twisted to form pre‐twisted strands. Subsequently, the four pre‐twisted strands are braided together to form a bundle. A copper wire is then helically wound around the braided bundle, which is coiled around a mandrel to produce a helical configuration. Finally, a thermal annealing process (conducted at 180 °C for 1 h) is applied to release the internal stresses and lock the helical configuration. Detailed descriptions and corresponding images are provided in Figure S2 (Supporting Information).

As a thermally driven actuator, BPAM can be controlled by its body temperature. To investigate its deformation characteristic, the contraction ratio of the BPAM is monitored over time at peak temperatures of 90 °C, 120 °C, and 150 °C, as shown in Figure 2b. The experimental results indicate that the contraction ratio gradually rises as the temperature increases, reaching 15.9% at 90 °C and approximately 41.0% at 150 °C. Furthermore, Figure 2c illustrates the length variations of BPAM under loads of 100 g, 200 g, and 300 g, with a target temperature set to 150 °C. The results demonstrate that BPAM exhibits a contraction ratio of 40.0% under a load of 300 g. Additionally, repeated heating‐cooling cycle tests show that the BPAM exhibits excellent deformation repeatability, as evidenced by stable contraction responses across multiple cycles.

Figure 2d illustrates the comparison between theoretical predictions and experimental results for length change with increasing temperature under loads of 100, 200, and 300 g, respectively. The results indicate that the predicted displacement trends closely match the experimental data, with a maximum error of 7.4% at a load of 200 g and only 2.2% at 300 g. The larger errors observed at 100 g are primarily due to contact between adjacent coils when the BPAM contracts below 50 mm in length. This contact restricts further contraction, resulting in the actual length being shorter than the prediction, which consequently increases the deviation. In contrast, under a load of 300 g, the coils remain separated throughout the contraction process, resulting in a closer agreement between the experimental and theoretical results.

To enable precise output force control of BPAM, we develop a closed‐loop system based on its temperature self‐sensing method and external force feedback. Experimental results demonstrate that the maximum deviation between the target and actual output forces is less than 0.06 N, occurring when the target force reached 3.5 N (see Figure 2e). The deviations of temperature and force throughout the actuation process are further presented in Figure S3 (Supporting Information). Furthermore, Figure 2f presents the measured stiffness of BPAM at various temperatures, along with the corresponding fitted curves. The results indicate a significant decline in stiffness with rising temperature, decreasing from 250 N m^−1^ at 30 °C to 75 N m^−1^ at 150 °C. Figure 2g demonstrates the target and actual temperatures of BPAM during the stiffness measurement experiments. The target temperature is set from 30 °C to 150 °C in increments of 30 °C, and the actual temperature quickly reaches the target value and remains stable until the target temperature changes. Meanwhile, the maximum steady‐state error for all target temperatures is 0.6 °C.

### 2.2 Design and Evaluation of the Silicone‐Coated BPAM for Underwater Actuation

**Figure 3:**
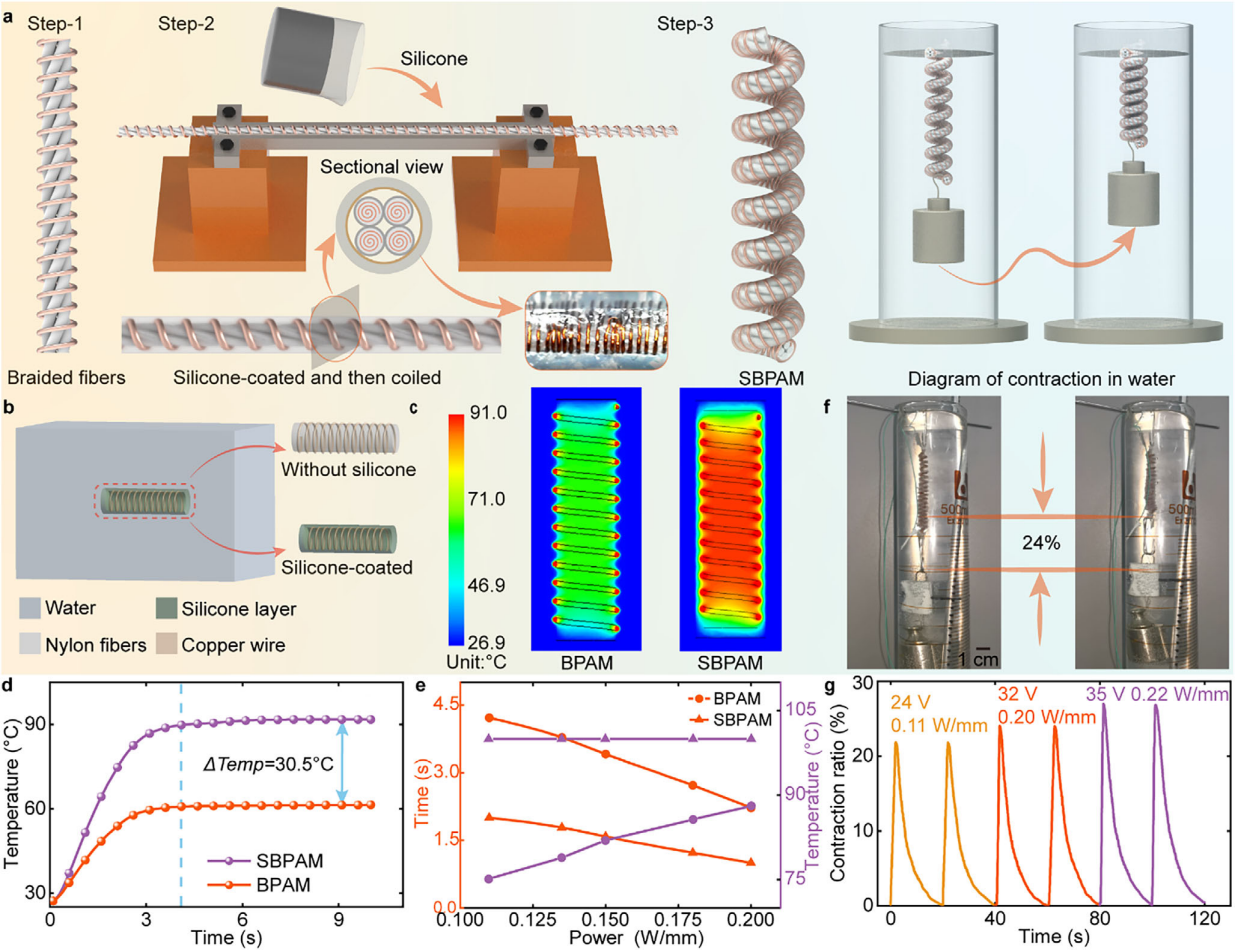
Development and thermal performance validation of SBPAM. a) Fabrication process and schematic diagram of SBPAM showing its contraction behavior in water. b) Simplified FEA model of BPAM and SBPAM used to simulate the heat transfer process, validating the effectiveness of the thermal insulation strategy inspired by the blubber layer of seals; the helical structure is unfolded into a straight segment for modeling. c,d) Temperature distributions of BPAM shown on the left and SBPAM on the right, highlighting a temperature difference of 30.5 °C at the fiber center under steady‐state conditions. e) Steady‐state temperature and required heating time of BPAM and SBPAM under identical input power. f,g) Contraction performance of SBPAM under different experimental conditions.

The application of BPAMs in underwater environments requires reducing thermal leakage. Drawing inspiration from the blubber layer of seals, a soft thermal insulation coating is applied to the surface of nylon fibers to inhibit direct convective heat transfer between BPAM and surrounding water. The fabrication procedure, depicted in **Figure** **3**a, consists of four main steps. First, a copper‐wire‐integrated fiber bundle is prepared and annealed in a vacuum oven. Next, the bundle is inserted into a thin tube with an inner diameter of 2 mm and mounted on a pedestal. Subsequently, silicone is injected to form an insulating layer approximately 0.2 mm in thickness. After complete curing, the coated fiber bundle is helically wounded on a mandrel and subjected to secondary thermal treatment at 180 °C for 1 h to stabilize the helical configuration. Finally, a novel BPAM capable of underwater actuation is obtained and referred to as the SBPAM.

The thermal insulation performance of the silicone insulation layer is investigated through finite element analysis (FEA), which compares the heat transfer behavior of BPAM in water, with and without the application of this layer. As illustrated in Figure 3b, the finite element model simplifies SBPAM by unfolding its helical structure into a straight segment, and incorporates a fluid domain to emulate the underwater environment. The SBPAM model is composed of nylon, copper, and silicone. The input temperature at the copper wire is programmed to rise from 25 °C to 100 °C within 2.1 s, and then held constant at 100 °C for 10 s. Figure 3c displays the temperature distributions of BPAM and SBPAM, highlighting the thermal difference at the center of the fiber. Notably, since the thermal conductivity of water is 24 times greater than that of air, the heat cannot be fully transferred to the center of the nylon fiber, resulting in the edge of the BPAM showing a higher temperature than the center in the water domain. The temperature difference reaches 30.5 °C under steady‐state conditions, confirming the insulation effectiveness of the silicone layer from a simulation perspective, as shown in Figure 3d.

The relationship between input power per unit length and the steady‐state temperature of BPAM and SBPAM is illustrated in Figure 3e. The steady‐state temperature of BPAM rises from 75 °C to 88 °C as the input power increases. In contrast, SBPAM reaches the target temperature of 100 °C under all tested input power conditions. In addition, the response time required to reach the steady‐state temperature is evaluated. When the input power is set to 0.11 W mm^−1^, SBPAM reaches a thermal steady state within 2.0 s, whereas BPAM requires 4.2 s. Collectively, these results highlight the improvement of thermal efficiency enabled by the silicone insulation layer. The contraction performance of SBPAM in water is characterized, as shown in Figure 3f,g. The heating time is increased to 2 s, and the cooling time is extended to 18 s to achieve the contraction to fully stabilize. The results indicate that SBPAM exhibits a stroke of 14 mm under an input power of 0.2 W mm^−1^. Moreover, we have conducted a durability test of SBPAM at an actuation frequency of 0.04 Hz (see Figure S9, Supporting Information). After 100 actuation cycles, the contraction ratio of SBPAM only slightly decreased from 23.6% to 21.4%, demonstrating excellent cyclic stability.

### 2.3 Development of a Rapid Actuation Unit Driven by SBPAMs

**Figure 4:**
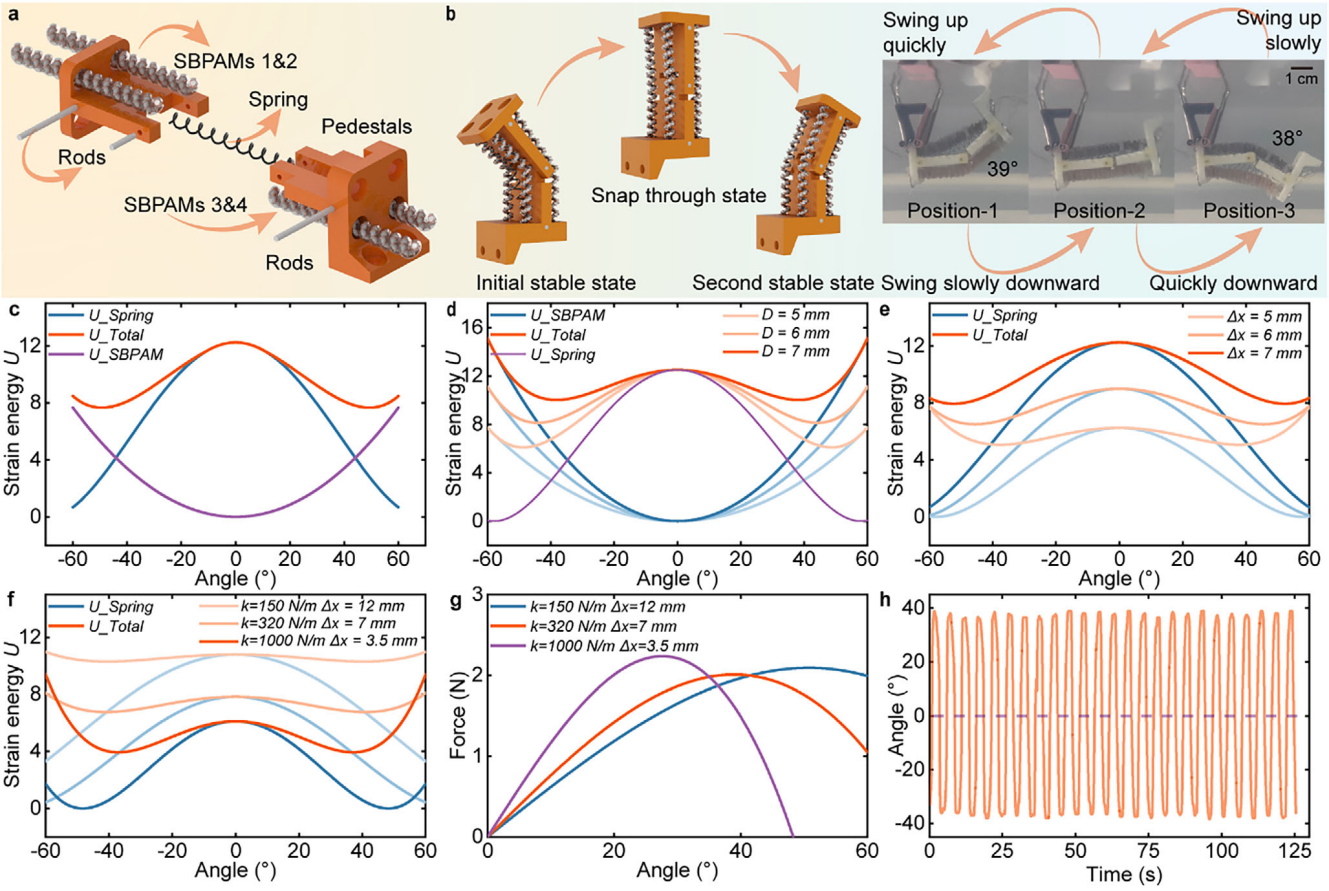
Development energy modeling of the RAU driven by SBPAMs. a) Exploded view of the RAU, which consists of SBPAMs, pedestals, rods, and a spring. b) Diagram of the rapid motion process of the RAU, showing two stable states and one snap‐through state. c) Variation of elastic potential energy of the RAU during transition between the two stable states. d–f) Elastic energy variation of the RAU under different structural parameters. g) Required output force of SBPAMs under three spring stiffness conditions. h) Angular displacement of the RAU at an actuation frequency of 0.2 Hz.

To improve actuation speed in underwater environments, a rapid actuation unit (RAU) driven by SBPAMs has been developed. It consists of two 3D‐printed pedestals, an energy storage spring, spring mounting rods, pedestal rods, and four SBPAMs (see **Figure** **4**a). The unit includes two bolt holes for integration with other components. Each side of the RAU is actuated by a pair of SBPAMs, which reduces the force required from a single SBPAM and lowers the temperature necessary for effective operation. The overall dimensions of the RAU are provided in Figure S4 (Supporting Information). This configuration improves energy efficiency and helps overcome the resistance posed by the underwater environment.

The motion of the RAU involves three sequential states: the initial stable state (Position‐1), the snap‐through state (Position‐2), and the second stable state (Position‐3), as shown in Figure 4b. This transition process can be better described by examining a single actuation cycle. Starting from the initial stable state, the two SBPAMs on the right contract, slowly swinging the pedestal upward toward the snap‐through state. During this phase, the spring elongates and stores elastic energy, while the pedestal moves gradually. Once the snap‐through state is reached, a sudden transition to the second stable state (Position‐3) is initiated. The elastic energy stored in the spring is rapidly released and transformed into kinetic energy, enabling a swift and forceful motion of the RAU.

The theoretical model is established to describe the relationship between the elastic potential energy of the RAU and its swing angle (see Figure S5 and Note S4, Supporting Information for details). The maximum output energy (*∆E*) is obtained by substituting the maximum swing angle (*θ*_s_) into the total elastic potential energy expression. Figure 4c presents the variations in the elastic potential energy of the SBPAMs, the spring, and the total elastic potential energy of the RAU. During the transition from Position‐2 to either Position‐1 or Position‐3, the elastic potential energy of the spring decreases, while the elastic potential energy of the SBPAMs increases, which causes a reduction in the total elastic potential energy. As the reduced elastic potential energy is efficiently converted into kinetic energy, the RAU achieves rapid motion. Meanwhile, when the RAU moves away from Position‐1 or Position‐3 without reaching Position‐2, it swings back and forth on one side. In this mode, the swing amplitude is smaller, but the frequency increases, a behavior referred to as monostable motion.

The key design parameters of the RAU are determined based on the previously established model. Figure 4d presents the variation in elastic potential energy when the distance between the SBPAMs and the neutral plane is set to 5 to 7 mm. The results indicate that a shorter distance leads to a larger difference in total system energy throughout the motion. Similarly, Figure 4e shows that an increase in the initial elongation of the spring leads to a larger difference in total energy. Furthermore, the stiffness and initial elongation of the spring should be considered in combination. Three representative configurations are evaluated: 1) *k* = 150 N m^−1^, *∆x*₀ = 12 mm, 2) *k* = 320 N m^−1^, *∆x*₀ = 7 mm, and 3) *k* = 1000 N m^−1^, *∆x*₀ = 3.5 mm. Figure 4f presents the variation in total elastic potential energy across the three conditions. The maximum energy values are 0.5, 1.15, and 2.2 mJ, corresponding to equilibrium angles of 42°, 39°, and 37°, respectively. The configuration with high stiffness and small initial elongation releases the greatest amount of energy. The maximum output forces in these conditions are 2.0, 2.1, and 2.2 N, with only minor variations, as shown in Figure 4g. Consequently, the spring with a stiffness of 1000 N mm^−1^ and an initial elongation of 3.5 mm is selected for the RAU.

The motion characteristics of the RAU are experimentally evaluated by alternately actuating the SBPAMs on both sides. Figure 4h shows that the transition from the snap‐through state to either stable state takes 0.2 s, corresponding to an angular velocity of ≈180° s^−1^. The RAU exhibits a swing angle range between −38° and +39°. Meanwhile, the cyclic performance test of the RAU has been conducted for 25 cycles (50 swing motions in total), which also showed good repeatability. These results validate the large‐amplitude, high‐speed actuation of the RAU driven by SBPAMs in underwater environments and highlight the significance of exploring the application of SBPAMs for underwater actuation.

### 2.4 Demonstration of the Bionic Ray

**Figure 5:**
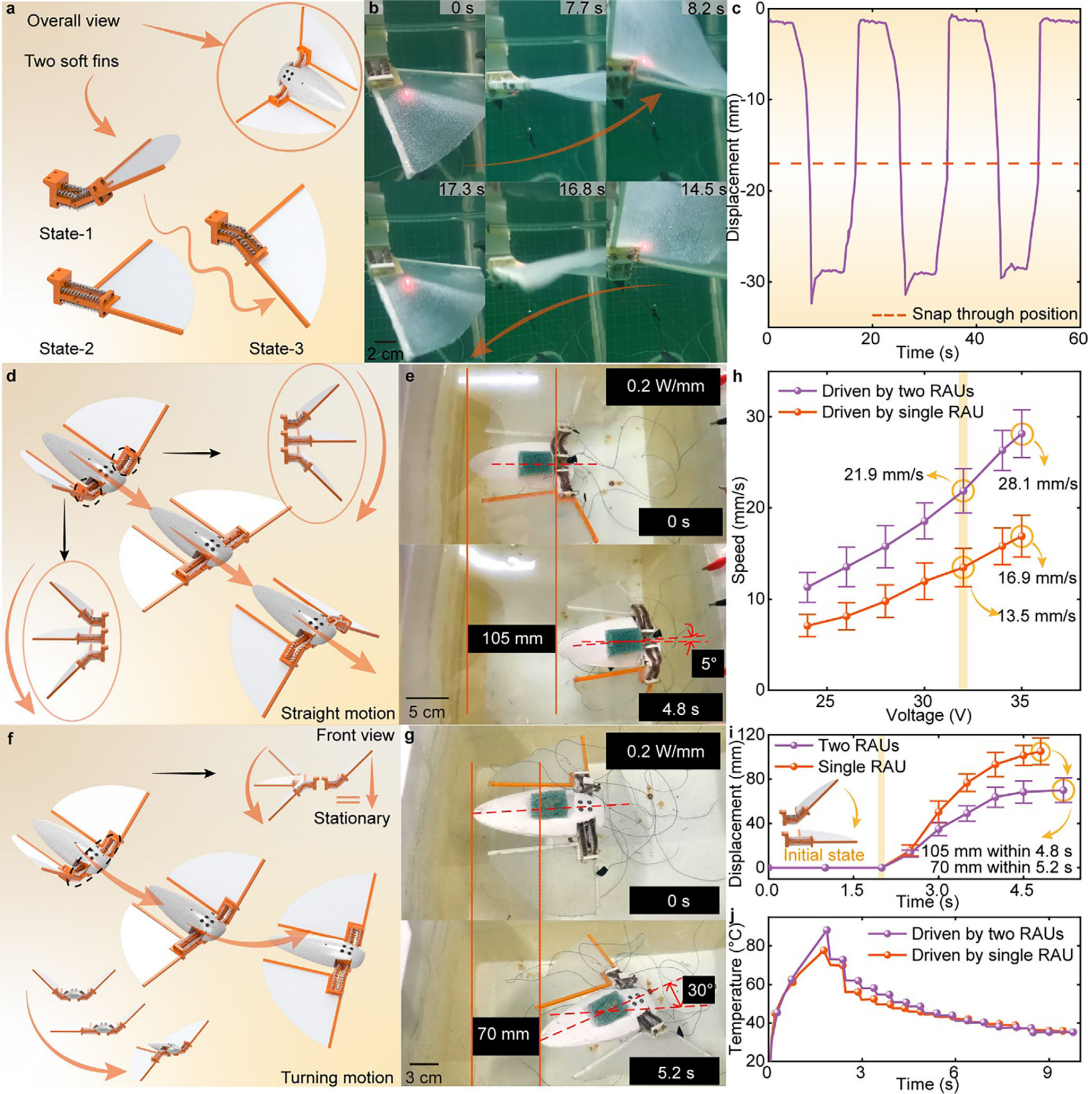
A bionic ray for demonstrating the underwater applicability of SBPAMs. a) Diagram of the soft pectoral fin securely integrated with the RAU. b,c) Displacement of the fin tip during a single actuation cycle. d–g) Straight and turning motions of the bionic ray, with a displacement of 105 mm and a turning angle of 30°. h,i) Swimming speed and per‐stroke displacement of the bionic ray under different actuation conditions; each data is tested three times (*n* = 3), and the mean and standard deviation are calculated to plot the error bars; j) Actuation temperature of the SBPAMs recorded during operation.

A bionic ray prototype is constructed to demonstrate the application of the proposed SBPAM in underwater actuation. It consists of a main body, two RAUs, and two soft pectoral fins, with the structural parameters illustrated in Figure S8 (Supporting Information). The soft fins are securely integrated with the RAUs, enabling passive, wave‐like deformation during actuation. The front section of the body is connected to the RAU pedestals, as shown in **Figure** **5**a. Figure 5b illustrates the flapping motion of a single fin, which closely replicates the movement of real rays. To evaluate the motion characteristics, the displacement of the fin is quantitatively measured, and the experimental setup is detailed in Figure 5c and Figure S6 (Supporting Information). Experimental results show that the fins pass through the snap‐through state and reach a stable state within 0.5 s, achieving a flapping angle of ≈25° and a peak angular velocity of 50° s^−1^. Additionally, the fin exhibits an amplitude of 28 mm and a tip displacement of 90 mm, which corresponds to 0.33 times the width of the bionic ray, closely matching the 0.35 ratio observed in real rays.

Figure 5d,e illustrates the schematic of the bionic ray during straight motion. When both pectoral fins flap simultaneously, the bionic ray swims forward, achieving a displacement of 105 mm with a deviation angle of 5° from the straight path. Figure 5f,g shows the turning motion of the bionic ray when only one pectoral fin is actuated and the other remains stationary. Under this condition, the ray turns toward the side opposite the active fin, resulting in a displacement of 70 mm within 5.2 s and a turning angle of 30°. These experiments confirm the bionic ray's ability to perform both straight and turning motions. The swimming speed is estimated from the displacement generated per stroke of the fin, as shown in Figure 5h. With both RAUs actuated simultaneously, the speed reaches 28.1 mm s^−1^. In contrast, when only one RAU is active, the speed drops to 16.9 mm s^−1^. Figure 5i,j summarizes the displacement per fin stroke and the corresponding actuation temperature of the SBPAM, respectively.

## 3 Discussion and Conclusion

This study investigates the potential of TAMs for underwater applications. However, due to the high thermal conductivity of water, rapid heat loss occurs during actuation, leading to reduced deformation and output force, and consequently suboptimal performance compared to actuation in air. To address these limitations, an optimized configuration of TAMs is proposed, integrated with a soft insulation layer to mitigate thermal loss. In addition, to enhance actuation speed, a rapid actuation unit based on elastic energy storage and release is developed and driven by the proposed SBPAMs. Furthermore, a bionic ray is constructed to demonstrate the underwater application of SBPAMs. The key contributions are summarized as follows.

Firstly, a theoretical model is established to describe the influence of structural parameters on the deformation and output force of TAMs. The deformation is positively correlated with the untwisting coefficient *c* and the mean diameter *D*, while the output force is proportional to the stiffness of the fiber bundle. To simultaneously achieve large deformation and high output force, the proposed BPAM draws inspiration from the climbing behavior of vine plants and applies three structural design strategies: adopting a helical configuration to enlarge the mean diameter, employing a pre‐twisting method to enhance the untwisting coefficient, and applying a multi‐fiber braiding approach to increase the stiffness and diameter of the fiber bundle. The BPAM achieves a contraction ratio of 40.0% under a load of 300 g by integrating these strategies. In future work, we will conduct multiple thermal cycles to assess the reproducibility and cycling stability of BPAM within a temperature range of 30 °C to 150 °C. This will facilitate an evaluation of the long‐term reliability of BPAM.

Secondly, to reduce thermal dissipation during underwater actuation, a thermal insulation strategy inspired by the blubber layer of seals is proposed, in which a silicone layer is coated onto the surface of the fiber. Finite element analysis is conducted to simulate the temperature distribution in the fiber during actuation. The results indicate a temperature difference of 30.5 °C at the center of the fiber between BPAM and SBPAM, validating the effectiveness of the insulation approach. The higher central temperature in SBPAM enables greater contraction performance compared to BPAM. In this work, the energy density, power density, and energy conversion efficiency of SBPAM under underwater conditions were quantitatively evaluated to assess its potential for practical applications. The calculated energy density and power density were 17.5 J kg⁻¹ and 8.75 W kg⁻¹, respectively. Further analysis revealed that the efficiency was 0.1% under an input power density of 0.1 W mm⁻¹ and decreased to 0.07% at 0.2 W mm⁻¹, while the specific energy consumption of SBPAM was 0.4 J mm⁻¹. The characteristics of BPAM, SBPAM, and other representative TAMs and soft actuators are systematically compared in **Table** **1** to provide a clearer perspective on their performance differences. The comparison includes materials, driving methods, diameter, contraction ratios and corresponding force, operation scenarios (air or water), response time, applied voltage, and energy conversion efficiency. It should be noted that such low efficiency is an inherent feature of thermally driven actuators, where substantial heat loss occurs through conduction, convection, and radiation to the surrounding water, particularly considering that the thermal conductivity of water is approximately 24 times higher than that of air. In addition, the energy conversion efficiencies of TAMs in air are also low, as reported by Xiang et al. (0.019%) and Noh et al. (0.075%), highlighting the trade‐off between large deformation capability and energy efficiency.^[^ ^33^, ^50^ ^]^

**Table 1:** Performance comparisons with some similar works.

First author	Materials	Driven methods	Diameter	Contraction ratio	Load force	Scenario	Response time	Voltage	Energy efficiency
Xiang et al.^[^ ^50^ ^]^	Nylon fiber and SMA	Thermal	1.5 mm	7.5%	300 g	Air	50 s	7.4 V	0.019%
Tang et al.^[^ ^53^ ^]^	Nylon and nickel fiber	Thermal	1.2 mm	12%	200 g	Air	5 s	5 V	0.049%
Noh et al.^[^ ^33^ ^]^	Polyethylene fibers	Thermal	1.5 mm	36	70 g	Air	20 s	N/A	0.075%
Hu et al.^[^ ^42^ ^]^	Carbon nanotubes	Thermal	0.11 mm	21.2%	15.6 Mpa	Air	0.5 s	N/A	N/A
Hu et al.^[^ ^48^ ^]^	Conductive polymer	Chemical	N/A	11%	5 Mpa	Air	50 s	0.4V	N/A
Zhou et al.^[^ ^49^ ^]^	Carbon nanotubes	Chemical	N/A	4%	5 Mpa	Air	25 s	0.8 V	N/A
This work	Nylon fiber and copper wires	Thermal	3.2 mm	40.0%	300 g	Air	50 s	12 V	0.29%
Chen et al.^[^ ^54^ ^]^	Liquid crystal elastomer	Thermal	6.3 mm	23%	0.2 N	Water	10 s	15.6 V	N/A
Li et al.^[^ ^55^ ^]^	DE and hydrogel	Electrical	116 mm	N/A	N/A	Water	0.14 s	6000 V	N/A
This work	Nylon fiber and copper wires	Thermal	3.4 mm	24%	100 g	Water	2 s	32 V	0.07%

Subsequently, a rapid actuation unit driven by SBPAMs is developed, enabling underwater swinging motion ranging from −38° to +39°, with an angular velocity of ≈180° s⁻¹. Finally, a bionic ray with two RAUs acting as pectoral fins is developed to demonstrate the underwater application of SBPAMs. With simultaneous flapping of both fins, the bionic ray achieves a displacement of 105 mm per stroke and a maximum swimming speed of 28 mm s^−1^. Moreover, the bionic ray is further compared with the existing underwater soft robotic fish. Yang et al.^[^ ^51^ ^]^ reported a soft fish actuated by liquid crystal elastomer (LCE) fibers with a speed of 15 mm s^−1^. Qing et al.^[^ ^52^ ^]^ introduced monostable soft flapping‐wing swimmers to achieve high‐speed locomotion. A common feature of both works is the use of high‐frequency monostable motion to generate continuous propulsion in robotic fish or swimmers. In contrast, our approach emphasizes powerful discrete flapping enabled by bistable elastic energy release, which is more compatible with the thermal characteristics of TAMs. This strategy is particularly suited for thermally driven artificial muscles, whose actuation frequency is fundamentally limited by the slow heat conduction to the center of fibers. In future work, we will focus on improving the actuation frequency and swimming performance of the bionic ray by reducing the thermal response time of SBPAM and optimizing the structural parameters of the RAU, such as spring stiffness and linkage length, to lower the required heating power while ensuring sufficient output force and deformation.

## 4 Experimental Section

### Materials and Simulation

The proposed BPAM and SBPAM are composed of nylon 6,6 fibers (each with a diameter of 0.5 mm), enameled copper wire, and silicone (0.2 mm in thickness). Finite element analysis (Ansys Workbench 2022) was performed to simulate Joule heat transfer from the enameled copper wire to the center of the nylon fiber under underwater conditions. The input temperature was applied to the copper wire, programmed to increase from 25 °C to 100 °C within 2.1 s and maintained at 100 °C for 10 s.

### Calculation Methods

The energy density, power density and energy conversion efficiency of SBPAM were calculated as follows: *energy* *density* = *W_out_*/*m_s_* = *m* × *g* × Δ*L*/*m_s_*, *power* *density* = *W_out_*/*m_s_*/*t* = *m* × *g* × Δ*L*/*m_s_*/*t*, and *energy* *efficiency* = *W_out_*/*W_in_* × 100%, where *W*_out_ is the mechanical energy output during contraction, *m* denotes the mass of the load, *m*_S_ denotes the mass of SBPAM, *g* is the gravitational acceleration, *ΔL* is the contraction displacement, *t* is the actuation time, and *W*_in_ is the input power.

### Experimental Methods

The flapping amplitude of the pectoral fin was tested by a laser displacement sensor (BX‐LV100N/R, Jingjiake Shenzhen), and the experimental setup was shown in Figure S6 (Supporting Information). Due to its limited measurement range and lack of waterproof capability, it was positioned above the water surface, and aligned with the outermost point of the RAU. The amplitude at the fin tip was then calculated through geometric conversion based on the known spatial relationship between the RAU and the fin, as shown in Figure S7 (Supporting Information).

### Statistical Analysis

All experiments were tested three times (*n* = 3), and the mean and standard deviation were calculated to generate error bars. Statistical analysis of the data was plotted by AxGlyph and Origin 2023.

## Conflict of Interest

The authors declare no conflict of interest.

## Author Contributions

J.S. and Y.F. contributed equally to this work. Y.L., J.L., and J.S. conceived this project. J.S. and Y.F. performed the finite element simulation. J.S. and Y.F. carried out experiments. J.S., Y.F., J.L., S.Z., and D.W. analyzed the experimental results and drew the figures. Y.L., J.S., and Y.F. wrote the draft. All authors participated in the discussions of the research.

## Supporting information



Supporting Information

Supplemental Movie 1

Supplemental Movie 2

Supplemental Movie 3

Supplemental Movie 4

Supplemental Movie 5

Supplemental Movie 6

## Data Availability

The data that support the findings of this study are available in the supplementary material of this article.
