# Supplementary material for: Bioinspired Twisted Artificial Muscles with Enhanced Performance for Underwater Applications
Source: Adv Sci (Weinh). 2025 Jul 11;13(15):e07572. doi: 10.1002/advs.202507572 (PMC13042987; doi:10.1002/advs.202507572)
Supplement: Supplementary file 1 — Supporting Information [file ADVS-13-e07572-s007.docx]

Supporting Information

**Bio-inspired Twisted Artificial Muscles with Enhanced Performance for Underwater Applications**

*Jin Sun#, Yuan Fu#, Shijing Zhang, Jing Li, Dehong Wang, Junkao Liu, Yingxiang Liu**

# *Jin Sun* and *Yuan Fu* contributed equally to this work

*Corresponding author. Email: liuyingxiang868@hit.edu.cn (Y.L.).

**The Supporting Information includes:**

**Note 1** Theoretical model of the BPAM.

**Note 2** Fabrication processes of BPAM.

**Note 3** Output force control of BPAM.

**Note 4** Rapid actuation unit (RAU) driven by SBPAM.

**Note 5** Measurement for the flapping amplitude of the fin.

**Note 6** Structural design and dimensional parameters of the bionic ray.

**Note 7** The cyclic performance testing of SBPAM.

**Figure S1** Force analysis of the helical structure.

**Figure S2** Fabrication processes of BPAM.

**Figure S3** Continuous monitoring of temperature and force deviation throughout the actuation cycle.

**Figure S4** Structural parameters of the RAU.

**Figure S5** The strain energy of the RAU and the required force of SB PAM.

**Figure S6** Experimental setup for measuring the flapping amplitude of the pectoral fin.

**Figure S7** Principle of amplitude measurement.

**Figure S8** Structural parameter of the bionic ray prototype.

**Figure S9** The durability test of SBPAM at an actuation frequency of 0.04 Hz.

**Other Supplementary Materials for this manuscript includes the following：**

**Movie S1** Fabrication process of BPAM.

**Movie S2** Actuation process of BPAM.

**Movie S3** Fabrication and actuation processes of SBPAM.

**Movie S4** Finite element simulation of temperature change in water.

**Movie S5** Configuration of the RAU driven by SBPAMs.

**Movie S6** Design and experiments of the bionic ray.

# Supplementary Notes

## Supplementary Note 1. Theoretical model of the BPAM

During the actuation process, the total deformation of BPAM includes two components: elastic deformation caused by elongation under axial load, and contractile deformation resulting from the untwisting torque generated within the twisted fibers upon temperature increase. In this work, a model correlating the structural parameters with contraction strain and output force of BPAM is established.

As shown in Figure S1, when a BPAM is subjected to an axial tensile force *F*, internal force components are generated on any normal cross-section of the twisted fiber. These include the axial force *F*_N_ perpendicular to the cross-section, the shear force *F*_Q_ parallel to the cross-section, the torsional moment *T* on the cross-section, and the bending moment *M* of the BPAM. Additionally, when the temperature of the BPAM increases, a thermally induced untwisting torque *T*_u_ also arises on the cross-section due to the fiber's thermal untwisting behavior. Therefore, when the BPAM contracts under a load force, the force analysis can be conducted on any normal cross-section. The corresponding force and moment components are described in Equation (1).

where *α* denotes the pitch angle of the BPAM，*D* is the diameter of the BPAM. When the axial external force *F* is set to a unit force, the internal forces on an arbitrary normal cross-section of the BPAM can be expressed as shown in Equation (2).

According to Mohr’s theorem, the deformation of the BPAM can be calculated as described in Equation (3).

where *E* is the elastic modulus of the twisted fiber; *A* is the cross-sectional area of the twisted fiber; *G* is the shear modulus of the twisted fiber; *I*_p_ is the polar moment of inertia of the fiber's cross-section; *I* is the moment of inertia of the fiber's cross-section; Δ*l* is the deformation of BPAM (mm); *l* is the original length of the fiber. Assuming the diameter of the fiber's cross-section is *d*, the following relationship can be derived.

The deformation of BPAM can be calculated as:

where *C*_1_ and *C*_2_ represent the load coefficient and the thermally induced untwisting torque coefficient, respectively, which are determined by the structural parameters of the BPAM and the material properties of the fiber, and can be expressed as:

where *N* denotes the number of the coils within BPAM. As mentioned before, the total deformation of the BPAM consists of two components: the elongation caused by the tensile load *F*, and the contraction induced by the thermally generated untwisting torque *T*_u_. A typical cross-section of the fiber is considered to analyze the effect of thermal untwisting torque. The temperature-induced untwisting generates torsional shear strain, which leads to corresponding shear stress and subsequently produces an untwisting torque. The specific relationship is defined in Equation (8).

where, Δ*ɸ* is the untwisting angle of the fiber caused by heating, *γ* is the torsional shear strain on the cross-section of the fiber, *τ* is the torsional shear stress on the cross-section of the fiber. The thermally induced untwisting torque can be obtained as:

By dividing the untwisting angle of the fiber caused by thermal activation by its length, a length-normalized untwisting angle is obtained. The relationship between this normalized untwisting angle and temperature is defined as untwisting coefficient *c*, as shown in Equation (11).

where *ΔT*_Temp_ denotes the temperature change. The expression for the thermally induced contraction of the BPAM can be derived, as shown in Equation (12).

In addition, the expression for the output force of the BPAM is derived. In practical applications, the output force of the artificial muscle is defined as the additional force required to prevent further contraction after it has contracted to a specific length *L*_pre_. According to Equation (5), the output force can thus be expressed as:

The stiffness of the BPAM can be obtained as:

where *ΔL*_1_ is the deformation of the BPAM before the load is applied, *ΔL*_2_ is the deformation of the BPAM after the load is applied. Consequently, the output force of the BPAM can be calculated as:

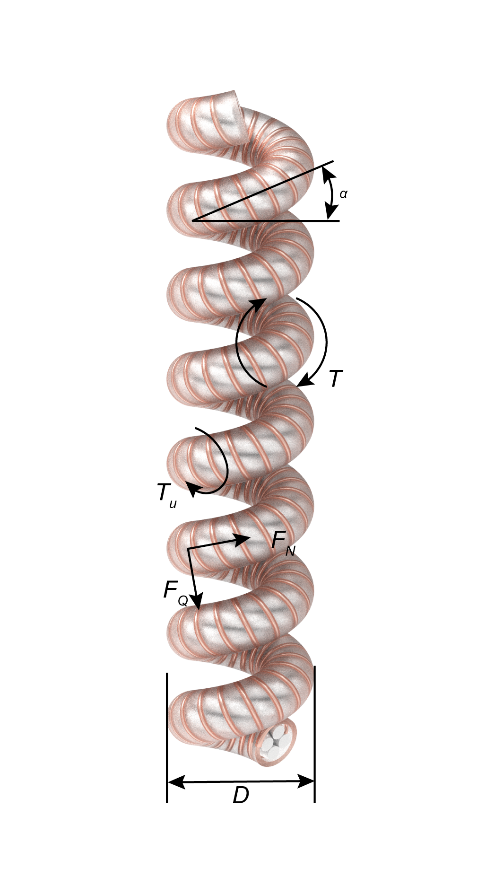


**Figure S1** Force analysis of the helical structure.

## Supplementary Note 2. Fabrication processes of BPAM

The fabrication process of the braided twisted artificial muscle (BPAM) is illustrated in **Figure** **S2**. One end of a single nylon fiber is fixed by a clamp, referred to as the fixed end. The other end is secured with a clip, which restricts its rotational motion while allowing axial displacement; this is referred to as the free end. A preload is applied to keep the fiber under tension during twisting. The motor twists the fiber through the clamp at the fixed end. Four nylon fibers with equal lengths and twist counts are first prepared, ensuring that the number of twists does not exceed a defined threshold. These fibers are then combined and twisted together into a bundle, with the total twist kept within a controlled range. The copper wire is wrapped around the bundle, which is then helically coiled around a mandrel with both ends fixed to prevent untwisting. Finally, the coiled structure undergoes thermal annealing through heating and slow cooling, resulting in a BPAM with stable structure.


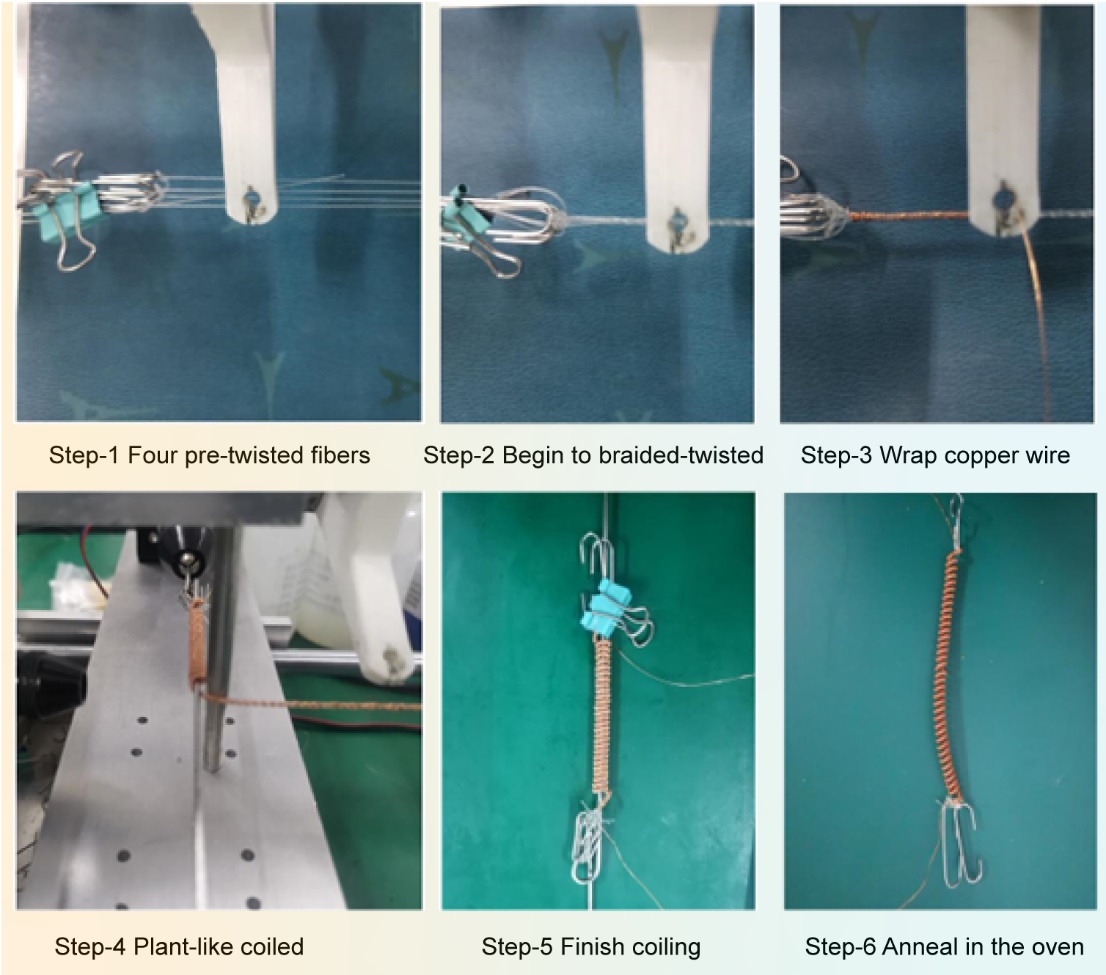


**Figure S2** Fabrication processes of BPAM.

## Supplementary Note 3. Output force control of BPAM

**Figure S3** presents the real-time deviations of both temperature and output force throughout the actuation cycle. The temporal temperature curve shows a controlled thermal response ranging from 40 °C to 95 °C. Simultaneously, the force deviation curve quantifies the tracking accuracy of the system, with the output force error remaining below 0.06 N throughout the operation. These results further confirm the stability and precision of the proposed output force control method.


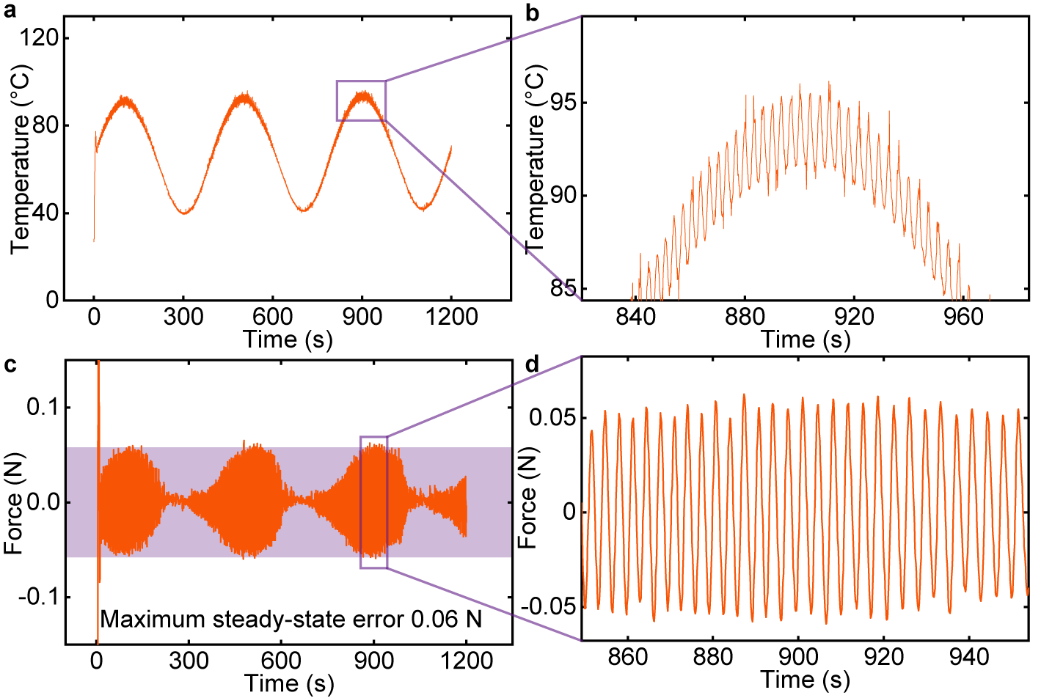


**Figure S3** Continuous monitoring of temperature and force deviation throughout the actuation cycle.

## Supplementary Note 4. Rapid actuation unit (RAU) driven by SBPAM

4.1 Structural parameters of the RAU

As shown in **Figure S4**, the three-view diagram of the rapid actuation unit (RAU) provides a detailed depiction of its structural composition. The RAU is composed of two 3D-printed pedestals, an energy storage spring, rods for spring fixation, pedestal rods, and four silicone-coated BPAMs. Each SBPAM is fixed at both ends using fine support rods, and the pedestals are assembled onto the rods using hot-melt adhesive.

The key geometric parameters of the RAU are as follows. These include the pedestal length is 25 mm, the distance from the spring fixation point to the pedestal rods is 20 mm, and the distance from the SBPAM to the central axis is 5 mm. The stiffness of the energy storage spring equals 1000 N/m with an initial extension 3.5 mm.


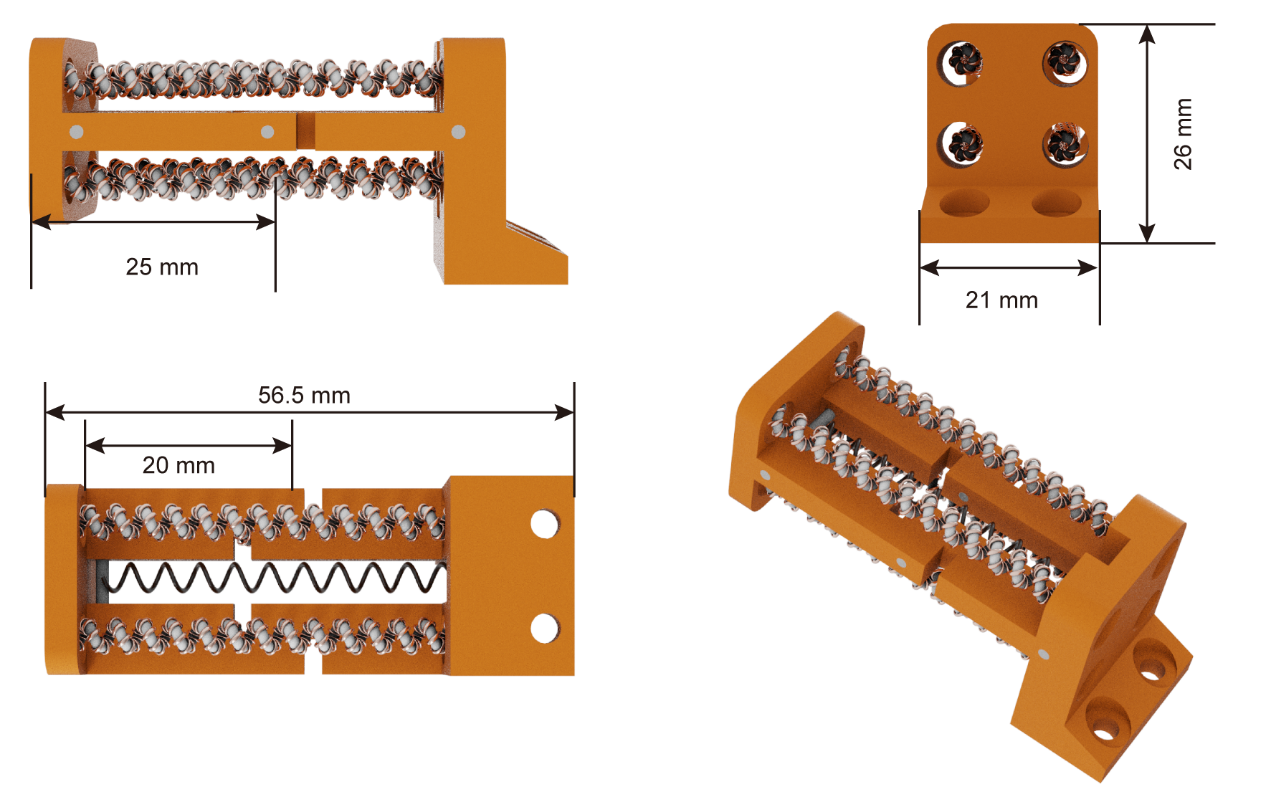


**Figure S4** Structural parameters of the RAU.

The durability and stability of unprotected springs over long periods of underwater operation is an important issue. This issue is directly related to the material of the spring, which is 304 stainless steel in this work. In addition, the evaluation can be further conducted in terms of both the water environment and the duration of immersion in water. The most common and economical choice for short-term application scenarios in freshwater is 304 stainless steel. However, for long-term immersion in contaminated freshwater, it is necessary to add a metallic coating such as electroplated zinc-nickel alloy or use 316 stainless steel to avoid rusting problems. Moreover, for applications in seawater, whether for short or long periods of immersion, it is essential to choose 316 stainless steel or even 2205 stainless steel. Therefore, the material of spring can be changed subsequently depending on the intended application.

4.2 Theoretical model of energy storage and required force of the RAU

The elastic potential energy of the system can be expressed as:

where *U_Total_*, *U_Spring_*, and *U_SBPAM_* denote the elastic potential energy of the system, the spring, and PTNA, respectively. Specifically, the elastic potential energy spring can be represented as:

where *k_s_* is the stiffness of the spring, *∆x_s_* denotes the stretch length of the spring and be obtained as:

where *x*_0_ is the pre-extension length of the spring, *l* is the length of the spring, and *θ* denotes the swing angle between different states.

Consequently, the change in elastic potential energy of the system can be represented as:

where *D* is the distance between SBPAM to the neutral surface of pedestal. We assume the lower half of the pedestal is fixed, and the force analysis is performed on the upper half of the pedestal, it can be described as:

where *M*_H_ is the is the resultant moment acting on the pedestal, *J* is the moment of inertia of the pedestal, *α* is the angular acceleration of the pedestal, *F*_T_ is the output force of the SBPAM, *F*_S_ is the tensile force of the spring which can be expressed as:

To initiate the swinging motion of the RAU, the output force generated by the SBPAM must result in a positive angular acceleration of the pedestal. Consequently, it must satisfy the condition：

**Figure S5a** presents the total strain energy of the RAU with spring stiffness values of 300 N/m, 600 N/m, and 900 N/m. It can be observed that the strain energy increases proportionally with the spring stiffness. **Figures S5b to S5d** illustrate the output force required from the SBPAM under different combinations of spring stiffness and pre-extension lengths. When the pre-extension length reaches 12 mm with a spring stiffness of 1000 N/m, the required force is as high as 13.95 N. This value decreases to 6.3 N and 2.2 N when the pre-extension lengths are reduced to 7 mm and 3.5 mm, respectively. Similarly, as the spring stiffness and pre-extension length decrease, the required output force is reduced; however, the total strain energy stored in the system also decreases accordingly.


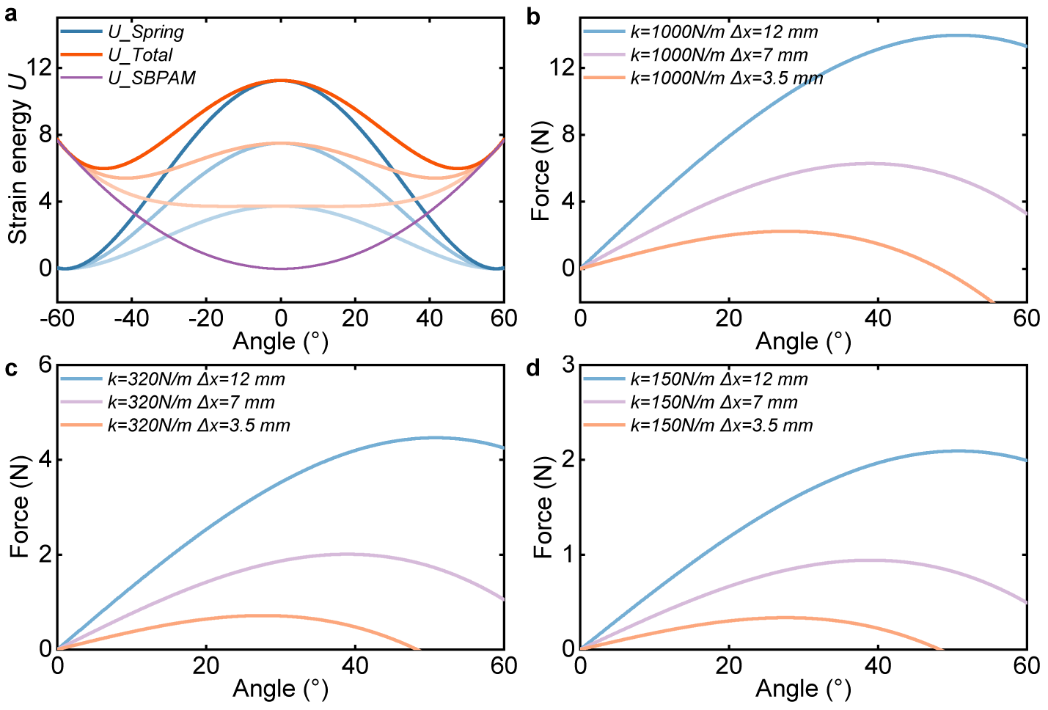


**Figure S5** The strain energy of the RAU and the required force of SBPAM. (A) The strain energy of the RAU with difference stiffness of the spring; (B-D) The required force of SBPAM under different stiffness and pre-extension length of the spring.

## Supplementary Note 5. Measurement for the flapping amplitude of the fin

The experimental setup for measuring the flapping amplitude of the pectoral fin is shown in **Figure S6.** A laser displacement sensor is used to measure the motion of the flapping unit. Since the sensor is not waterproof, it is positioned above the water surface, with the laser beam aimed at the RAU. Due to its limited measurement range, which is shorter than the distance to the tip of the pectoral fin, the laser is aligned with the outermost point of the RAU. The amplitude at the fin tip is then calculated through geometric conversion based on the known spatial relationship between the RAU and the fin, as shown in **Figure S7**.


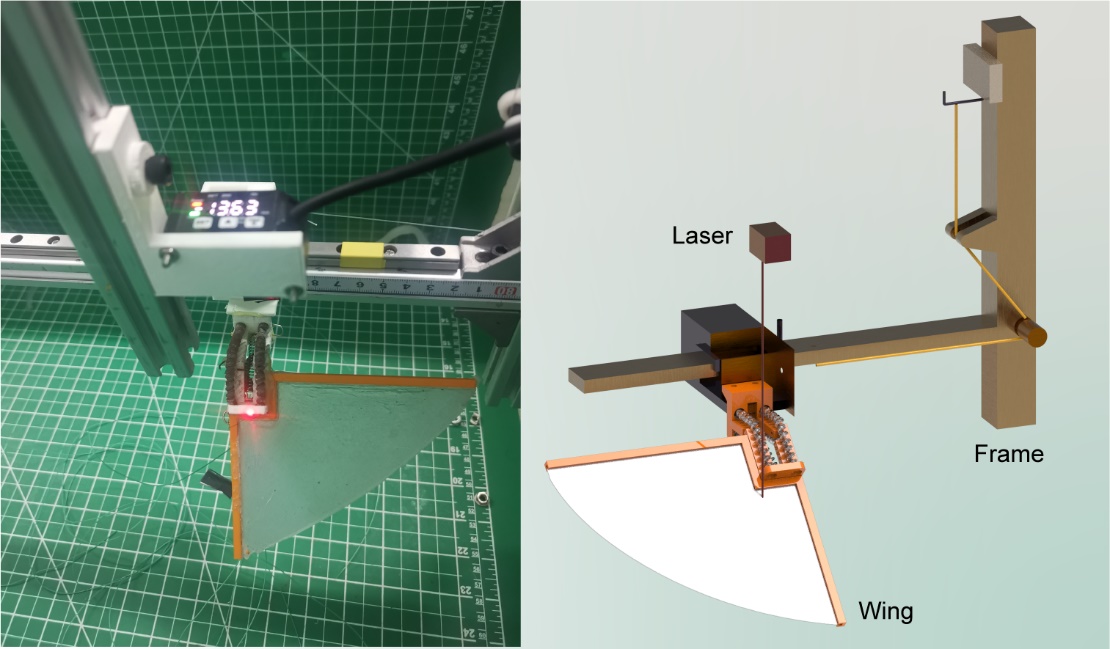


**Figure S6** Experimental setup for measuring the flapping amplitude of the pectoral fin.


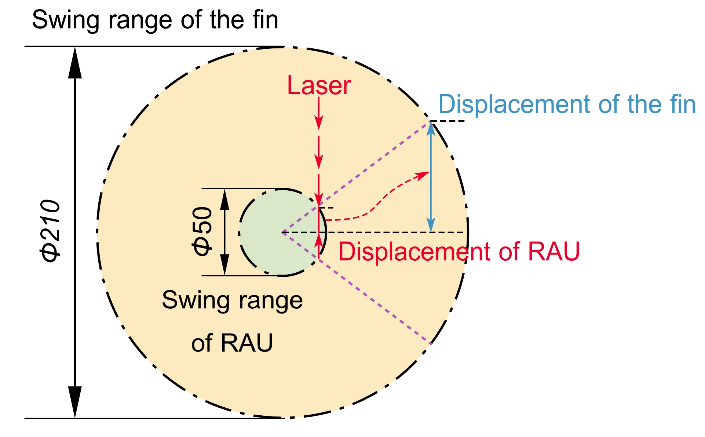


**Figure S7** Principle of amplitude measurement.

## Supplementary Note 6. Structural design and dimensional parameters of the bionic ray

Based on the RAU thickness of 26 mm, the body thickness of the bionic ray is set to 28 mm. Referring to the NACA 0020 airfoil profile, the body length is determined to be 140 mm, as shown in **Figure S8**. The side view reveals that any cross-section along the body follows the NACA 0020 profile. The front view shows that each cross-section is also streamlined and laterally flat, ensuring streamlined performance in both forward swimming and turning directions while reducing viscous drag in the surrounding fluid. The structural parameters of real rays are referenced in the design, including body aspect ratio, fin aspect ratio, and fin-to-body area ratio. Based on the determined body length, the final dimensions of the pectoral fins and the prototype are established. The lengthwise length of the pectoral fins is 127 mm, the widthwise length is 105 mm, and the overall width of the bionic ray is 276 mm, with a total body length of 140 mm. The proportions of the prototype closely resemble those of real rays, ensuring the structural rationality of the bionic design.


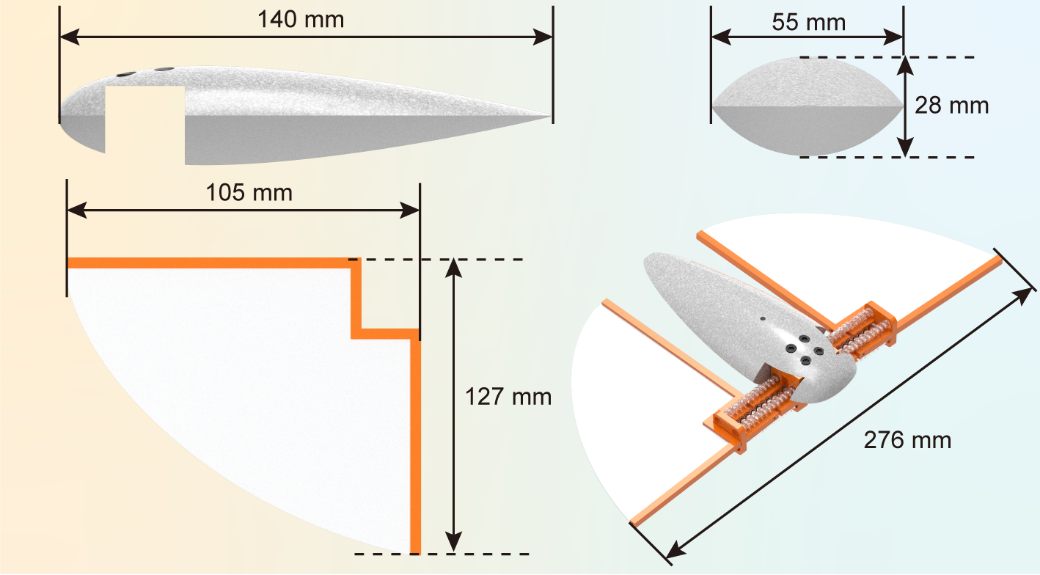


**Figure S8** Structural parameters of the bionic ray prototype.

## Supplementary Note 7. The cyclic performance testing of SBPAM

The complete actuation cycle of the SBPAM consists of two processes: heating for contraction and cooling for elongation. The long heat dissipation time for cooling to room temperature limits the operating frequency of SBPAM in air. On the other hand, when the actuator is driven in water, the thermal conductivity of water is greater than that of air. This results in faster heat dissipation and cooling, which enhances the operating frequency through accelerated cooling and heat dissipation. In contrast, the proposed thermal insulation strategy aims to increase the heating rate of the SBPAM in water, and the operating frequency can be increased by reducing the heating time. The preliminary durability test of SBPAM at an actuation frequency of 0.04 Hz is conducted. As shown in **Figure S9**, after 100 actuation cycles, the contraction ratio of SBPAM decreased from 23.6% to 21.4%, demonstrating excellent cyclic stability and reliability.

**
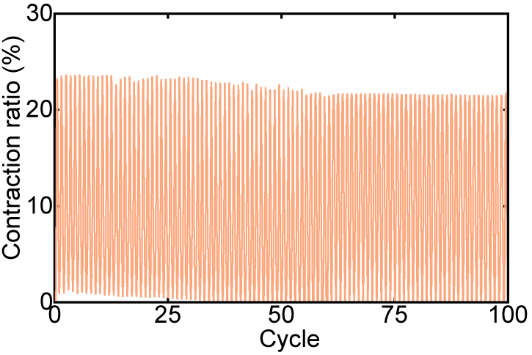
**

**Figure S9** The durability test of SBPAM at an actuation frequency of 0.04 Hz.
